# Vaccine-Associated Thrombocytopenia and Thrombosis: Venous Endotheliopathy Leading to Venous Combined Micro-Macrothrombosis

**DOI:** 10.3390/medicina57111163

**Published:** 2021-10-26

**Authors:** Jae C. Chang, H. Bradford Hawley

**Affiliations:** 1 Department of Medicine, University of California Irvine School of Medicine, Irvine, CA 92868, USA; 2 Department of Medicine, Wright State University Boonshoft School of Medicine, Dayton, OH 45435, USA; hbhawley@twc.com

**Keywords:** combined micro-macrothrombosis, COVID 19 vaccines, endotheliopathy, immune thrombocytopenic purpura-like syndrome, multiorgan dysfunction syndrome, thrombotic thrombocytopenic purpura-like syndrome, venous endotheliopathy-associated vascular microthrombotic disease

## Abstract

Serious vaccine-associated side effects are very rare. Major complications of vaccines are thrombocytopenia and thrombosis in which pathogenetic mechanism is consistent with endotheliopathy characterized by “attenuated” sepsis-like syndrome, leading to the activation of inflammatory and microthrombotic pathway. In the COVID-19 pandemic, acute respiratory distress syndrome caused by microthrombosis has been the major clinical phenotype from the viral sepsis in association with endotheliopathy-associated vascular microthrombotic disease (EA-VMTD), sometimes presenting with thrombotic thrombocytopenic purpura (TTP)-like syndrome. Often, venous thromboembolism has coexisted due to additional vascular injury. In contrast, clinical phenotypes of vaccine complication have included “silent” immune thrombocytopenic purpura (ITP-like syndrome), multiorgan inflammatory syndrome, and deep venous thrombosis (DVT), cerebral venous sinus thrombosis (CVST) in particular. These findings are consistent with venous (v) EA-VMTD. In vEA-VMTD promoted by activated complement system following vaccination, “consumptive” thrombocytopenia develops as ITP-like syndrome due to activated unusually large von Willebrand factor (ULVWF) path of hemostasis via microthrombogenesis. Thus, the pathologic phenotype of ITP-like syndrome is venous microthrombosis. Myocarditis/pericarditis and other rare cases of inflammatory organ syndrome are promoted by inflammatory cytokines released from activated inflammatory pathway, leading to various organ endotheliitis. Vaccine-associated CVST is a form of venous combined “micro-macrothrombosis” composed of binary components of “microthrombi strings” from vEA-VMTD and “fibrin meshes” from vaccine-unrelated incidental vascular injury perhaps such as unreported head trauma. This mechanism is identified based on “two-path unifying theory” of in vivo hemostasis. Venous combined micro-macrothrombosis due to vaccine is much more serious thrombosis than isolated distal DVT made of macrothrombus. This paradigm changing novel concept of combined micro-macrothrombosis implies the need of combined therapy of a complement inhibitor and anticoagulant for CVST and other complex forms of DVT.

## 1. Introduction

Although serious vaccine complications are rare, thrombocytopenia, cerebral venous sinus thrombosis (CVST), and inflammatory organ syndrome, such as myocarditis and pericarditis following COVID-19 vaccination, are well documented [1,2]. However, their pathogenetic mechanisms are poorly understood. In COVID-19 viral sepsis, acute respiratory distress syndrome (ARDS) is caused by complement-activated endotheliopathy leading to inflammation and vascular microthrombotic disease (VMTD) as previously analyzed [3]. Similar to vaccine, thrombocytopenia, deep vein thrombosis (DVT) and inflammation also have occurred in COVID-19 sepsis. The clinical features of ARDS, laboratory evidence of partial hemostasis, and pathologic phenotype of microthrombosis have been well-established to be caused by endotheliopathy triggering molecular pathogenesis [3,4]. Endotheliopathy promotes exocytosis of ULVWF multimers, which activates ULVWF path of hemostasis that leads to formation of microthrombosis and consumption of platelets leading to the thrombocytopenia. In addition, unexpected thrombosis oftentimes coexisted with microthrombosis presenting as macrothrombotic disease (i.e., venous thromboembolism (VTE)), which was likely caused by virus-unrelated additional vascular injury such as vascular access, device, procedure, surgery, or use of mechanical ventilation in hospital/ICU admitted patients [4]. This macrothrombotic disease has confounded clinicians in the understanding of its relationship to the underlying microthrombosis.

Thrombocytopenia and macrothrombotic disease of CVST after vaccination and those of VTE in COVID-19 sepsis are very similar in hemostatic features each other. Further, VTE also has occurred in vaccinated patients and CVST has developed in COVID-19 sepsis, and inflammation has been common finding in both. In view of similar clinical phenotypes of thrombosis, thrombocytopenia and inflammation occurring in both COVID-19 infection and vaccine complication, the pathogenesis of vaccine complication is suspected to be the result of a shared mechanism of endotheliopathy in sepsis. In this article, we will analyze the characteristic clinical and laboratory data in vaccine-induced hemostatic disease focusing on endothelial molecular pathogenesis utilizing “two activation theory of the endothelium”.

## 2. Endotheliopathy

Complement activation initiated by a pathogen leads to endotheliopathy of the host that causes a hemostatic disease promoted by microthrombogenesis, leading to damage to the endothelial cells (ECs) [3,5]. Endotheliopathy activates two simultaneous but independent pathogenetic processes: inflammatory pathway and microthrombotic pathway. Each pathway promotes separate characteristic molecular pathogenesis. The former releases inflammatory cytokines such as interleukin 6, tumor necrosis factors, and interferons, and the latter promotes exocytosis of unusually large von Willebrand factor (ULVWF) multimers that initiate lone activation of ULVWF path of hemostasis [5]. Thus, sepsis is commonly manifested by inflammation, and endotheliopathy-associated VMTD (EA-VMTD) expressed as disseminated microthrombosis, which orchestrates thrombotic thrombocytopenic purpura (TTP)-like syndrome with the triad of consumptive thrombocytopenia, microangiopathic hemolytic anemia (MAHA) and hypoxic multiorgan dysfunction syndrome (MODS) [6].

Inactivated or killed pathogen vaccines, their subunits, or toxoid vaccines have long been theorized to promote pathogen-specific protective antibody production and T cell immune response against each specific pathogen [7], but also were sometimes associated with undesired vaccine-associated enhancement of viral infections [8] or “attenuated” sepsis-like syndrome. Indeed, the clinical features of thrombocytopenia, thrombosis, and inflammatory organ syndrome after COVID-19 vaccination have been similar to that of COVID-19 sepsis which causes inflammation and VMTD [9,10,11]. COVID-19 typically presents with endotheliopathy with microvascular microthrombosis in the lungs, causing ARDS, and occasionally thrombocytopenia, MAHA, and other organ syndromes, which have been called TTP-like syndrome [3,4]. However, vaccine-associated endotheliopathy does not cause TTP-like syndrome, but instead is manifested as “silent” immune thrombocytopenic purpura (ITP) [12,13], which can be called ITP-like syndrome. In addition, CVST [14,15] and multiorgan inflammatory syndrome (MOIS) [16,17] have occurred after vaccination. The distinguishing clinical features of endotheliopathy-associated VMTD (EA-VMTD) between viral sepsis and vaccine complication can be explained by vascular physiologic, hemodynamic and histological differences of the vascular wall between the arterial system and venous system (Table 1), which include oxygen delivery function, pressure difference, shear stress, and vascular and microvascular networks. COVID-19 sepsis is consistent with combined venous and arterial endotheliopathy (i.e., venous EA-VMTD [vEA-VMTD] and arterial EA-VMTD [aEA-VMTD]) and the vaccine complication is exclusively congruous with the effect of venous endotheliopathy (i.e., vEA-VMTD).

The clinical features of the serious complication of COVID-19 vaccines are consistent with “venous endotheliopathy” leading to vEA-VMTD, which is characterized by ITP-like syndrome, inflammatory organ syndrome, and a complex form of DVT. In contrast, COVID-19 sepsis, in addition to venous endotheliopathy, may also present with clinical features of “arterial endotheliopathy” leading to aEA-VMTD, which has been demonstrated by TTP-like syndrome, hypoxic MODS and arterial thrombosis/gangrene syndrome such as limb gangrene and symmetrical peripheral gangrene [4]. COVID-19 sepsis also has been associated with complex forms of DVT, such as VTE, pulmonary thromboembolism (PTE), and splanchnic vein thrombosis (SVT). These coexisting venous thrombotic syndromes and arterial endotheliopathy are consistent with the concept that COVID-19 sepsis promotes both aEA-VMTD and vEA-VMTD. The pathogenesis of complex forms of COVID-19-associated arterial and venous thrombotic syndromes has been identified by applying the “two-path unifying theory” of hemostasis on the formation of combined micro-macrothrombi [4]. This extraordinary mechanism will be elaborated later. Table 1 shows why vaccine complications are consistent with venous endotheliopathy (vEA-VMTD).

## 3. Thrombocytopenia

**Figure 1:**
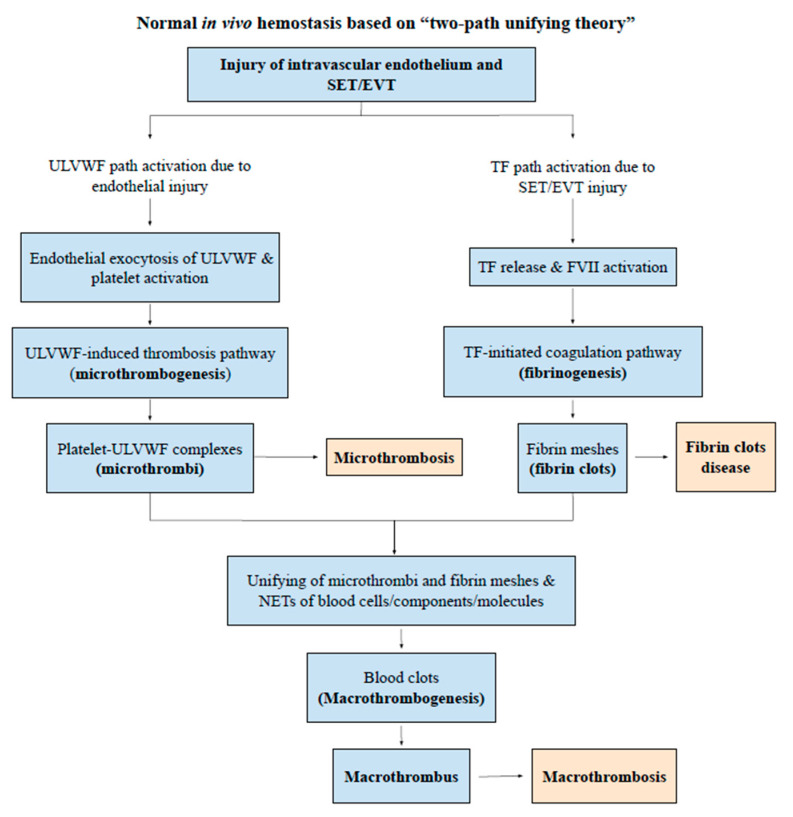
Normal in vivo hemostasis based on “two-path unifying theory” (Reproduced and modified with permission from Chang JC. Thrombosis Journal. 2019;17:10). Legends/Captions: Following a vascular injury, in vivo hemostatic system activates two independent sub-hemostatic paths: microthrombotic (ULVWF) and fibrinogenetic (TF). They are initiated by the damage of ECs and/or SET/EVT in a external bodily injury and intravascular injury. In the activated ULVWF path from ECs damage, ULVWF multimers are released and recruit platelets, and produce microthrombi strings via microthrombogenesis, but in the activated TF path from SET/EVT damage, released TF activates FVII and produces fibrin meshes via extrinsic coagulation cascade. The final path of in vivo hemostasis is macrothrombogenesis, in which microthrombi strings and fibrin meshes become unified together with incorporation of NETs, including red blood cells, neutrophils, DNAs and histones. This unifying event macrothrombogenesis promotes hemostatic plug formation and wound healing in external bodily injury and produces macrothrombosis in intravascular injury. Abbreviations: EA-VMTD, endotheliopathy-associated vascular microthrombotic disease: EVT, extravascular tissue; NETs, neutrophil extracellular traps; SET, subendothelial tissue; TF, tissue factor; ULVWF, unusually large von Willebrand factor.

Thrombocytopenia is a very common complication in sepsis and other critically ill patients. As shown in novel “two-path unifying theory” of hemostasis (Figure 1), it occurs due to platelet consumption as a consequence of microthrombogenesis. Microthrombi are produced via endothelial molecular pathogenesis following activation of ULVWF path of hemostasis [5]. The “microthrombi strings” are composed of platelet-ULVWF complexes anchored to the endothelial membrane. It occurs in the capillaries and arterioles in aEA-VMTD and perhaps in smaller veins in vEA-VMTD. In the past, when the mechanism of consumptive thrombocytopenia was not recognized, it had been called “thrombocytopenia in critically ill patients” (TCIP) [18].

**Figure 2:**
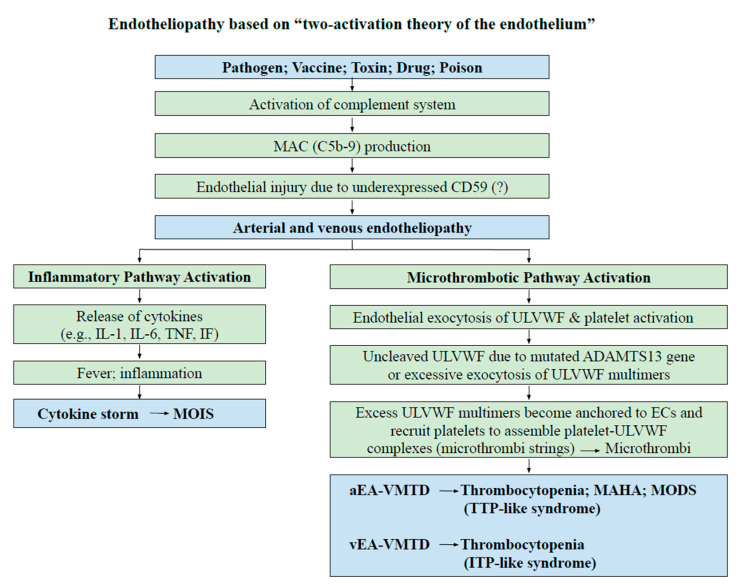
Endotheliopathy based on “two-activation theory of the endothelium”. Endothelial molecular pathogenesis is initiated by activation of complement system triggered from pathogen, vaccine, toxin, drug, poison, or others. However, unlike pathogen-induced sepsis, vaccine-induced clinical syndrome is almost always due to venous endotheliopathy because it has produced ITP-like syndrome rather than TTP-like syndrome occurring in arterial endotheliopathy as explained in the text. These distinguishing features are caused by differences in physiological function and hemodynamic characteristics between the arterial system and venous system. Therefore, MAHA and hypoxic MODS do not occur in vaccine-associated endotheliopathy. However, inflammatory syndromes (MOIS) such as myocarditis and pericarditis could occur after vaccination instead of hypoxic MODS. Abbreviations: ITP, immune thrombocytopenic purpura; MAC, membrane attack complex; IF, interferon; IL, interleukin; MAHA, microangiopathic hemolytic anemia; MODS, multiorgan dysfunction syndrome; MOIS, multiorgan inflammatory syndrome; TNF, tumor necrosis factor; TTP, thrombotic thrombocytopenic purpura; aEA-VMTD, arterial endotheliopathy-associated vascular microthrombotic disease; vEA-VMTD, venous endotheliopathy-associated vascular microthrombotic disease; ULVWF, unusually large von Willebrand factor multimers.

**Figure 3:**
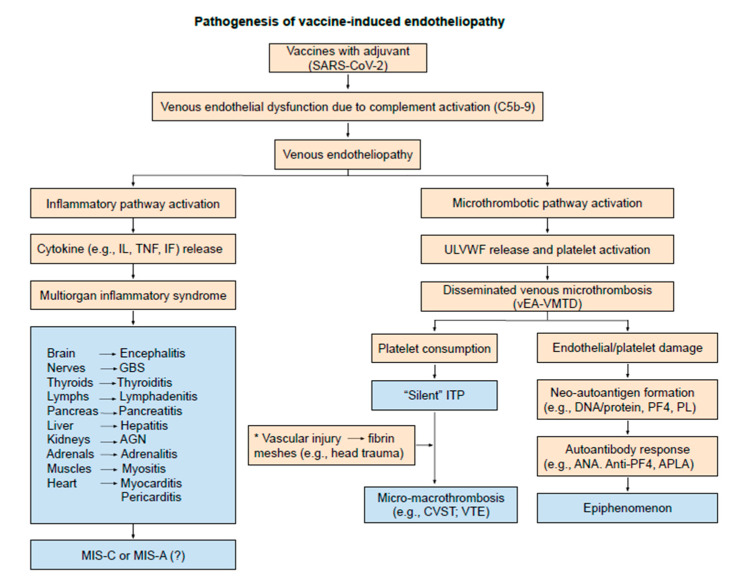
Pathogenesis of vaccine-induced endotheliopathy. The venous endothelial pathogenesis in SARS-CoV-2 vaccines is self-explanatory. Compared to aEA-VMTD/vEA-VMTD of sepsis, vEA-VMTD from vaccination does not typically cause MAHA and life-threatening hypoxic MODS. However, it can cause its share of another combined micro-macrothrombotic syndrome, a complex form of DVT, especially CVST as explained in the text. Incidentally, the occurrence of VTE in sepsis and CVST in vaccination provides additional insights in thrombogenesis for the important role of vascular accesses in the hospitalized patient, ICU in particular. Sub-references for observed MOIS shown in Table S1 are available in Supplementary file. Abbreviations: aEA-VMTD, arterial endotheliopathy-associated VMTD; vEA-VMTD, venous EA-VMTD; APLA, antiphospholipid antibodies; PF4, platelet factor 4; PL, phospholipids; CVST, cerebral venous sinus thrombosis; DVT, deep venous thrombosis; ITP, immune thrombocytopenic purpura; MAHA, microangiopathic hemolytic anemia; MIS-C, multisystem inflammatory syndrome in children; MIS-A, MIS in adults; MODS, multiorgan dysfunction syndrome; MOIS, multiorgan inflammatory syndrome; VTE, venous thromboembolism; AGN, acute glomerulonephritis; GBS, Guillain-Barre syndrome: IF, interferon, IL, interleukin; TNF, tumor necrosis factor.

Since both sepsis and vaccination are associated with aEA-VMTD and/or vEA-VMTD, thrombocytopenia following vaccination is obviously due to consumption from microthrombogenesis. To date, the pathogenesis of ITP, both idiopathic type and immune-designated type, has been considered to be due to immune destructive mechanism of platelets [19,20,21,22]. In the COVID-19 pandemic, thrombocytopenia occurring in vaccine-associated thrombosis has been called “COVID-19 vaccine-associated (immune) thrombosis and thrombocytopenia (VITT)” [1,23,24], often related to platelet factor 4 (PF4) antibodies [25,26]. However, no clear cause-effect relationship has been established [27] between thrombocytopenia and macrothrombosis, as well as thrombocytopenia and anti-PF4 antibodies. Indeed, the mechanism of thrombocytopenia, either platelet consumption or destruction in vaccine-associated thrombocytopenia and thrombosis, has not been determined. The concept of ITP cannot be defined properly without comprehension of “endothelial molecular pathogenesis”. Figure 2 illustrates the pathogenesis of endotheliopathy based on “two-activation theory of the endothelium” [18], and the “two-path unifying theory” of hemostasis is shown in Figure 1 [28]. Based on these theories, we have also formulated the pathogenesis of vaccine-associated endotheliopathy”, as illustrated in Figure 3.

The pathogenesis presented in Figure 3 is logical and straightforward. Vaccine-associated venous endotheliopathy activates two pathogenetic pathways. One is the inflammatory pathway promoting MOIS such as myocarditis, pericarditis, thyroiditis [29,30], lymphadenitis [31,32] and others as exampled in Table S1, and the other is the microthrombotic pathway promoting ITP-like syndrome of microthrombosis in the venous system. ITP-like syndrome may be “calm before storm” because it can be transformed itself into deadly combined micro-macrothrombosis (e.g., CVST) if complicated by additional vascular injury (e.g., incidental head injury following vaccination) as pointed in Figure 3.

Perhaps, following mild venous endotheliopathy after vaccination, most of microthrombi strings could rapidly undergo physiologic microthrombolysis by protease ADAMTS13, which results in the breakdown of platelets, as well as ECs and ULVWF multimers. This microthrombolysis can lead to release of platelet antigens such as PF4, phospholipids (PL), and other proteins which may become neo-autoantigen and produce anti-PF4 antibodies [25,26] and anti-PL antibodies [33]. There is no proof that thrombocytopenia is the result of platelet destruction due to anti-PF4 antibodies or anti-PL antibodies. Furthermore, solitary thrombosis (e.g., distal DVT) formation is a local or regional hemostatic process and requires only small amounts of platelets without consequence of thrombocytopenia. On the contrary, thrombocytopenia in TTP-like syndrome, ITP-like syndrome, and TCIP in bacterial and viral sepsis is not of immunologic mechanism, but due to consumption of platelets in the process of microthrombogenesis triggered by generalized endotheliopathy [18].

Thus, in the early phase of viral sepsis and vaccination, positive anti-PF4 antibodies and anti-PL antibodies as well as antinuclear antibodies, and anti-dsDNA antibodies observed in endotheliopathy are likely an “epiphenomenon” unrelated to thrombosis [27,34,35]. Additionally, most cases of chronic and acute ITP are suspected to be the phenotypes of vEA-VMTD, which also explains the paradox of thrombophilic state producing complex forms of DVT in patients with ITP. This mystery has been articulated by many concerned hematologists [36,37,38]. The intriguing relationship between vEA-VMTD (i.e., ITP-like syndrome) and thrombophilic state of macrothrombosis (i.e., DVT) will be addressed in the heading of the “pathogenesis of venous combined micro-macrothrombotic syndromes” derived from the unifying mechanism of hemostasis [28].

## 4. Thrombosis

The term “thrombosis” has been used to denote every “blood clot(s)” in circulation as a generic disease entity in both arterial and venous systems. According to contemporary dogma, “thrombosis” in vivo is produced via extrinsic cascade progression from TF release out of subendothelial tissue (SET) to activation of coagulation factors, involving various serine proteases, protein substrates and fibrinogen, and to formation of fibrin meshes/fibrin clot(s). However, this simple concept of thrombogenesis based on TF activating extrinsic coagulation system alone cannot define the clinical and pathological aspects of many different thrombotic disorders observed in clinical medicine and pathology. Examples are:

- Macrothrombosis includes DVT, VTE, CVST, PTE, splanchnic vein thrombosis (SVT) and Budd-Chiari syndrome, portal vein thrombosis, acute ischemic stroke, acute myocardial infarction, aortic thrombus and renal vein thrombosis, etc.
- Microthrombosis (i.e., VMTD) occurs in TTP, TTP-like syndrome, ARDS, diffuse encephalopathic stroke, microvascular myocardial infarction, hemolytic-uremic syndrome, transient ischemic attack, microaneurysm and thrombosis of retinal artery, MODS, and hepatic veno-occlusive disease.
- Heparin-induced thrombocytopenia with thrombosis syndrome, especially white clot syndrome, is a unique aberrant hemostatic disease without vascular injury.
- Gangrene syndrome associated with arterial combined micro-macrothrombosis includes symmetrical peripheral gangrene (SPG), purpura fulminans, Fournier’s gangrene in acute promyelocytic leukemia (APL), Burger’s disease, diabetic gangrene, Raynaud’s phenomenon, and acute necrotizing fasciitis.
- Fibrin clot disease occurs in APL as aberrant hemostatic disease without vascular injury.
- Acute “disseminated intravascular coagulation (DIC)” is a form of VMTD with hemorrhagic complication.
- Concurrent microthrombosis and macrothrombosis in both arterial and venous systems in paroxysmal nocturnal hemoglobinuria (PNH) is an unresolved disease yet.

Certainly, many different forms of thrombosis do occur resulting from complexity of hemostatic mechanism affected by: (1) two sub-hemostatic paths (i.e., ULVWF and/or TF); (2) their unifying path to macrothrombogenesis; (3) venous or arterial systems; (4) microvasculature or larger vasculature; (5) localization in different vascular trees (i.e., localized and generalized, and solitary and multiple); and (6) altered hemostatic components associated with mutated genes (e.g., protein C, protein S, FV Leiden, and ADAMTS13, etc.). These variable combinations produce different forms of thrombi which vary in the size, number, tissue and organ location, and vascular system affected and determine many different clinical phenotypes [39,40]. Therefore, the inclusively designated term “thrombosis” cannot provide clinically explicative diagnosis for each thrombotic disorder because the term thrombosis only means an “existential” blood clotting disorder [39,41].

For example, although distal DVT and proximal/central(catheter) DVT are recognized as the same character disease of macrothrombus in the venous system, these two disorders are suspected to be caused by very different molecular composites expressing as two dissimilar clinical, pathological and radiological phenotypes (Table 2). Likewise, the pathologic phenotypes of acute ischemic stroke in the arterial system and CVST in the venous sinus occur as the result of macrothrombus, but their clinical phenotypes are different not only because of their location in the brain and blood vessel but also because of their character of the blood clot. On the other hand, the clinical phenotype of CVST stroke may be very similar to diffuse encephalopathic stroke, their pathologic phenotypes are distinctly different because the former is due to localized venous macrothrombosis, but the latter is due to disseminated microvascular microthrombosis [40].

## 5. Cerebral Venous Sinus Thrombosis

Now, how did CVST, which is a form of DVT, develop as macrothrombosis in both sepsis due to a pathogen and vaccine as a side effect?

There are two different pathogenetic phenotypes of DVT. One is from trauma resulting in local injury that causes localized solitary/distal DVT composed of fibrin clot (macrothrombosis). The other is vascular injury-induced DVT in a hospitalized patient with sepsis or coexisting underlying disease or conditions, such as diabetes, hypertension, atherosclerosis, pregnancy and cancer, or in a vaccinated individual with ITP-like syndrome (e.g., vEA-VMTD). In the latter situations, microthrombosis might have already existed, but was further complicated by additional localized vascular damage provoking micro-macrothrombosis. These underlying microthrombi and newly formed fibrin meshes can unify together in circulation and produce a localized or regional DVT complex (i.e., proximal/central DVT) producing combined micro-macrothrombosis characterized by multiple, large, extended, irregular and connected or disconnected binary micro-macrothrombi complexes. Distal DVT is a benign disease, but proximal/central DVT is a life-threatening malignant disease. The pathogenetic mechanism of CVST occurring in sepsis or following vaccination is promoted by the same mechanism of unifying process of micro-macrothrombogensesis, but in vaccinated individuals it is more likely occurring in the outpatient setting. In those individuals, CVST could have been due to unreported incidental head injury [42], which is a not uncommon trauma in life.

CVST has been very rare in both COVID-19 infection and post-vaccination, but it is seen more commonly than other DVT among the non-hospitalized vaccinated individuals. On the other hand, VTE and other DVT have more commonly occurred than CVST among hospitalized COVID-19 infected patients, in which cases vascular access injury such as surgery could have played a major role. Therefore, exclusive occurrence of CVST following outpatient vaccination might have been related to unidentified head injury. Indeed, following COVID-19 vaccines severe headache was present with thrombocytopenia prior to the diagnosis of CVST in some patients [43], which suggests unreported incidental head trauma might have preceded CVST and underlying ITP-like syndrome might have preexisted. To understand the mystery of combined micro-macrothrombosis manifesting as a phenotype of complex DVT as well as CVST, the two basic concepts of hemostasis should be applied; one is the mechanism of preexisting microthrombogenesis (i.e., underlying venous endotheliopathy), and the other is the “unifying mechanism” of “microthrombi” from vEA-VMTD and “fibrin meshes” from local vascular injury as follows.

## 6. Pathogenesis of Venous Combined Micro-macrothrombotic Syndromes

The normal unifying mechanism between microthrombi strings and fibrin meshes following a local vascular injury (e.g., aortic aneurysm with intraluminal thrombosis and distal DVT) is a localized natural process of hemostasis as shown in Figure 1 [28,39]. However, the same unifying process of “microthrombi strings” from septic/vaccine endotheliopathy and “fibrin meshes” from vascular injury in or outside of the hospital, leading to combined micro-macrothrombosis, seems to be a far-fetched proposition. However, its pathogenetic mechanism logically explains clinical and laboratory findings of each of the arterial and venous combined micro-macrothrombotic syndromes [4,5], as detailed in Table 3 and Table 4. Arterial combined micro-macrothrombosis leads to gangrene syndromes (e.g., SPG), but venous micro-macrothrombotic syndrome leads to complex DVT (e.g., VTE). These phenotypes display striking clinical/radiological imaging features with poor prognostic outcome and raise serious issues in clinical management.

In the venous system, prior to vascular injury vEA-VMTD (i.e., “silent” ITP/ITP-like syndrome) could have already existed due to an underlying disease, such as diabetes or hypertension, or COVID-19 vaccines could have initiated venous endotheliopathy leading to “silent” microthrombosis. This ITP-like syndrome can be transformed to venous combined micro-macrothrombosis such as CVST when fibrin meshes are released into cerebral venous sinus circulation due to head trauma even in an outpatient setting, as noted in Figure 3. The results of the unified micro-macrothrombi in venous sinuses of the vaccinated individuals would be less dramatic in clinical phenotype than that of gangrene type in arterial circulation because of two reasons in the venous system; first, blood circulation is afferent toward the heart so that combined micro-macrothrombi present in blood returning to the heart do not cause tissue and organ hypoxia/anoxia; second, because the unifying process of “fibrin meshes” and “microthrombi strings” is slow and continuous with low shear stress flow, it does not produce minute macrothrombi, but forms large, multiple, irregular, extended. and connected or disconnected striped blood clots made of micro-macrothrombi complexes at vicinity of the venous injury. This mechanism produces complex forms of DVT in different locations (e.g., VTE, PTE, SVT, CVST, PVT, BCS, etc). Although the binary character of the micro-macrothrombi complex is the same to that of arterial circulation, different physiologic and hemodynamic nature between arterial and venous systems assembles completely different clinical phenotypes of “micro-macrothrombi” listed in Table 3. Summarized in Table 4 is a diagnostic guide for vaccine-associated vEA-VMTD and it also identifies major clinical components in differential diagnosis from sepsis-associated aEA-VMTD.

Fortunately, the vaccinated individual avoids the risk of in-hospital vascular injury, which is thought be the reason why, instead of VTE, CVST is more prevalent after vaccination, likely due to incidental head injury which may be the main risk factor in the outpatient setting. However, if a vaccinated CVST patient is admitted to the hospital, the risk for other complex forms of DVT is expected to rise due to inherent nature of vascular events in the hospital, and in the ICU in particular.

## 7. Molecular Evidence of Venous Endotheliopathy

EA-VMTD resulting from the pathogenesis of COVID-19 sepsis-associated endotheliopathy can be manifested by clinical phenotypes of ARDS and MODS, pathologic phenotype of microthrombosis, and laboratory findings of TTP-like syndrome (i.e., consumptive thrombocytopenia and MAHA) in rare cases and ITP-like syndrome in many cases. Furthermore, EA-VMTD in COVID-19 sepsis has been characterized by elevated endothelial markers (i.e., increased activity of FVIII, and overexpressed ULVWF/VWF antigen), and decreased ADAMTS13 activity [4]. Thus, coexisting VTE and PTE can be inferred as combined micro-macrothrombosis resulting from underlying ITP-like syndrome of vEA-VMTD and virus-unrelated another vascular injury. These multiple clinical phenotypes are consistent with the proposition that COVID-sepsis causes both aEA-VMTD and vEA-VMTD. On the other hand, limited clinical features of vaccine-associated endotheliopathy to ITP-like syndrome and CVST are consistent with vEA-VMTD.

In the medical literature, CVST cases that occurred in pre-pandemic era were characterized by markedly overexpressed endothelial markers of VWF antigen and activity [44,45,46,47] and increased activity of FVIII [44,45,48,49,50,51,52], just as in COVID-19 sepsis-associated CVST. These findings certainly confirm that venous endotheliopathy existed as the underlying pathology of ITP-like syndrome prior to occurrence of post-vaccine CVST. These abnormal endothelial markers should be confirmed from every CVST patients with vaccine-associated thrombocytopenia.

Separately, as previously mentioned, thrombocytopenia in the vaccinated patients is due to consumption of platelets, and positive anti-PF4 antibodies and anti-PL antibodies as well as antinuclear antibody and anti-dsDNA antibody are more consistent with epiphenomenon of venous endotheliopathy unrelated to thrombocytopenia or thrombosis including CVST. The epiphenomenon was likely a secondary event following endothelial cell and platelet protein damage. This epiphenomenon is one of the lessons we have learned from COVID-19 sepsis [53]. In retrospect, some of the vaccinated individuals had ITP-like syndrome and CVST was formed as venous combined micro-macrothrombosis following additional cerebral venous sinus injury [54].

Complement activation plays the key role in vaccine-induced endotheliopathy, contributing to the endothelial molecular pathogenesis [55,56,57,58]. In addition, certain adjuvants and surfactants conjugated with vaccines trigger complement activation and causes injury to the endothelium, especially if an individual is mild to moderately deficient in ADAMTS13, which may promote venous endotheliopathy. In both COVID-19 sepsis and vaccination, the ligand spike (S) protein from SARS-CoV-2 has been thought to interact with the ACE2 receptor on the endothelial cells (ECs) of the host and activate complement system [59] and produce C5b-9 complex, which can lead to endotheliopathy. It promotes both inflammation and microthrombogenesis by releasing inflammatory cytokines and ULVWF multimers [16,60], as seen in sepsis. The adjuvants and surfactants polyethylene glycol and polysorbate 80, and graphene oxide sometimes used in vaccines are known to trigger complement activation [61,62,63]. These chemicals turn on the complement when they are attached to molecules, especially lipids. The size of the adenovirus delivered lipid coated particle is larger than the mRNA lipid coated particle, which may more likely lead to complement activation [64].

## 8. Diagnostic and Therapeutic Consideration

### 8.1. Diagnostic Perspective

The earliest indicator suggestive of venous endotheliopathy following vaccination is asymptomatic thrombocytopenia (ITP-like syndrome). CVST or DVT presenting with obvious macrothrombosis and persistent inflammation should alert the possibility of serious complication of vaccination. The diagnosis of venous combined micro-macrothrombosis can be established with the laboratory findings of (1) thrombocytopenia; (2) overexpressed ULVWF/VWF antigen or activity; (3) increased activity of FVIII; (4) markedly increased D-dimers; and (5) increased thrombomodulin [65], as shown in Table 4. Thrombocytopenia is the result of platelet consumption and is not due to immune destruction. Overexpressed ULVWF/VWF antigen, increased activity of FVIII and thrombomodulin in CVST are endothelial markers released from endotheliopathy, support on-going microthrombogenesis resulting in ITP-like syndrome, but are not the indicators of hypercoagulable state [5]. Markedly increased D-dimers and positive fibrin degradation products are the results of activated TF path supporting the diagnosis of combined micro-macrothrombosis [4]. The imaging study of venous combined micro-macrothrombotic syndrome may show as large, multiple, irregular, connected and extended, or disconnected macrothrombosis at the region of vascular injury and travelling afferently to the heart.

### 8.2. Therapeutic Approach

Since vaccine-induced venous endotheliopathy is triggered by the activation of the complement system, the best therapeutic approach would be anti-complement therapy that can suppress both inflammation and microthrombogenesis promoting ITP-like syndrome [4].

“Silent” ITP/ITP-like syndrome (i.e., vEA-VMTD) may be treated with an available antimicrothrombotic regimen of therapeutic plasma exchange or N-acetyl cysteine to counteract venous microthrombogenesis or anticomplement therapy of intravenous immunoglobulins (IVIG) to stimulate the complement attenuation by amplifying C3 convertase level or act as a receptor for activated complement components [66,67]. Uncomplicated ITP-like syndrome should not be treated with platelet transfusion alone because it will increase platelet consumption and may even promote formation of complex forms of DVT when prolonged intravenous line is used for therapeutic plasma exchange, IVIG or platelet transfusion [68,69].

Theoretically, the best approach for preventing CVST and VTE in the general population is not to vaccinate individuals with a condition or existing disease known to cause microvascular microthrombosis due to endotheliopathy, such as pregnancy, diabetes, uncontrolled hypertension, or autoimmune disease. If vaccinated, the individual should be alerted for the risk of “blood clots” and be instructed to avoid every circumstance of prolonged vascular access and any injury, such as head trauma, for a duration of 6 to 8 weeks. Clinical trials are needed for the treatment of venous combined micro-macrothrombosis utilizing antimicrothrombotic agent and anticoagulant.

Complement activation also plays a key role in vaccine-associated MOIS. If inflammatory syndrome(s), such as myocarditis, pericarditis, or Guillain-Barre syndrome, is overlapped with ITP-like syndrome, IVIG and steroid therapy would be appropriate to prevent endothelial dysfunction and support endothelial integrity [70].

### 8.3. “Special Note” on TTP-Like Syndrome and ADAMTS13 after COVID-19 Vaccines 

In the pre-COVID-19 pandemic era, only a handful of cases of TTP-like syndrome had been recorded following vaccination [71]. Questionable cases of vaccine-associated TTP-like syndrome or relapsed TTP have been reported [72,73]. To the best of our knowledge well-documented case of de novo TTP after vaccination has not been documented after COVID-19 vaccines, but latest two cases of TTP-like syndrome after COVID-19 vaccine may support the relationship between COVID-19 vaccine and arterial endotheliopathy [74,75]. Since TTP-like syndrome (aEA-VMTD), which has often been associated with ADAMTS13 insufficiency and arterial endotheliopathy, it is possible that additional components of vaccines such as surfactants or adjuvants in vaccines might cause some effects on the arterial system via complement activation. In both aEA-VMTD and vEA-VMTD, ADAMTS13 insufficiency is a thrombophilic condition via the activated ULVWF path due to its heterozygous gene mutation/polymorphism or imbalanced/relative deficiency caused by excessive exocytosis of ULVWF multimers in endotheliopathy [5]. Therefore, in patient care and vaccine development, the medical community should be vigilant with the close monitoring of TTP-like syndrome and ITP-like syndrome and evaluate the role of ADAMTS13 for the diagnostic use and therapeutic potential [4,5,6].

## 9. Conclusions

The pathogenesis of vaccine-associated thrombocytopenia is characterized by venous endotheliopathy promoting microthrombosis and platelet consumption (ITP-like syndrome) triggered by an activated complement system due to inactivated pathogen/toxin/viral molecule and/or adjuvant conjugated with vaccine. When venous microthrombosis of vEA-VMTD encounters fibrin meshes from an activated TF path following vaccine-unrelated vascular injury, VTE or CVST occurs as a result of the unifying mechanism of microthrombi and fibrin meshes, leading to venous combined micro-macrothrombosis. The clinical hemostatic disorders and inflammatory syndromes are consistent with attenuated sepsis-like syndrome. Fortunately, arterial endotheliopathy has not been a major issue after COVID-19 vaccination.

## Figures and Tables

**Table 1:** Clinical phenotypes and mechanisms of endotheliopathy in arterial and venous systems per “two-activation theory of the endothelium”.

Clinical Phenotype	Arterial Endotheliopathy	Venous Endothelipathy
**Underlying pathology**	aEA-VMTD	vEA-VMTD
Physiological/hemodynamic differences in the vascular network	Efferent circulation from the heart (oxygenated blood delivery to the organs)	Afferent circulation into the heart (deoxygenated blood delivery to the lungs)
	High pressure flow	Low pressure flow
	High shear stress	Low shear stress
	Capillary and arteriolar microvascular event	Venous and pulmonary microvascular event
Primary cause and result		
Vascular injury (ECs)	Sepsis-induced microvascular endotheliopathy	Sepsis-induced venous endotheliopathy
		Vaccine-induced venous endotheliopathy
Vascular pathology site	Disseminated aEA-VMTD at the microvasculature	Transient or “silent” vEA-VMTD at venous system
Activated hemostatic path	ULVWF path	ULVWF path
Thrombosis component	Microthrombi strings	Microthrombi strings
Clinical phenotypes	TTP-like syndrome - consumptive thrombocytopenia - MAHA - MODS/MOIS	ITP-like syndrome - consumptive thrombocytopenia - Absence of hemolysis - MOIS




Abbreviations: aEA-VMTD, arterial endotheliopathy-associated vascular microthrombotic disease; vEA-VMTD, venous-VMTD; ECs, endothelial cells; ITP, immune thrombocytopenic purpura; MAHA, microangiopathic hemolytic anemia; MODS, multiorgan dysfunction syndrome; MOIS, multiorgan inflammatory syndrome; TTP, thrombotic thrombocytopenic purpura; ULVWF, unusually large von Willebrand factor.

**Table 2:** Two faces of deep venous thrombosis with pathologic, pathogenetic and laboratory differences based on two-path unifying theory of hemostasis and endothelial molecular pathogenesis.

Phenotypes	Distal DVT (de novo Venous Macrothrombosis)	Proximal/Central DVT (Combined Venous Micro-Macro thrombosis)
Disease examples		
Venous thrombosis		
DVT	Distal DVT	Proximal/central(catheter) DVT
VTE		VTE; multiple PTE
Other complex venous thrombosis		IVCT; SVT; PVT; BCS; SVCT; CVST
**Mechanisms of vascular injury**		
Event	Local trauma (rarely with surgery/vascular access)	Underlying disease (vEA-VMTD (e.g., sepsis)) + local trauma (commonly with surgery/vascular access)
Extent of vascular damage	Local ECs/SET injury	Disseminated ECs injury + local/regional ECs/SET injury
**Pathogenesis**		
Activated thrombosis path	ULVWF and TF paths from local trauma	ULVWF path from systemic vEA-VMTD and TF path from regional trauma
Thrombi character	Macrothrombus	Combined micro-macrothrombi composed of “microthrombi strings–fibrin meshes”
Severity	Typically, solitary, benign, and self-limited	Serious and often with multiple/large thrombi
Severe inflammation	Absent	May be present and can be severe
Venous EA-VMTD	Absent	Commonly present (e.g., ITP-like syndrome)
MOIS	Absent	May be present
**Diagnostic findings/markers**		
Consumptive thrombocytopenia	Does not occur	Sometimes occurs
ULVWF/VWF antigen	Normal	Overexpressed
FVIII activity	Normal	Increased
ADAMTS13 activity	Normal	Mild to moderately decreased
D-dimer	Normal	Markedly increased
Immune: ANA; APLA; Anti-DNA antibodies	Negative	May be positive
**Therapeutic approach per theory**	No treatment or short-term anticoagulant	Anticoagulant and antimicrothrombotic/anticomplement agent (?)

Abbreviations: ANA, antinuclear antibodies; APLA, antiphospholipid antibodies; BCS, Budd-Chiari syndrome; CVST, cerebral venous sinus thrombosis; DNA, deoxyribonucleic acid; DVT, deep vein thrombosis; vEA-VMTD, venous endotheliopathy-associated vascular microthrombotic disease; ECs, endothelial cells; EVT, extravascular tissue; ITP, immune thrombocytopenic purpura; IVCT, inferior vena cava thrombosis, MOIS, multiorgan inflammatory syndrome; PTE, pulmonary thromboembolism; PVT, portal vein thrombosis; SET, subenthelial tissue; SVCT, superior vena cava thrombosis; SVT, splanchnic vein thrombosis; TF, tissue factor; ULVWF, unusually large von Willebrand factor; vEA-VMTD, venous endotheliopathy-associated vascular microthrombotic diseases; VTE, venous thromboembolism; VWF, Von Willebrand factor.

**Table 3:** Spectrum of combined micro-macrothrombotic syndromes.

Pathology/Phenotypes	Arterial Micro-Macrothrombotic Syndrome (aEA-VMTD)	Venous Micro-Macrothrombotic Syndrome (vEA-VMTD)
**Primary pathogenesis**		
Cause example	Pathogen-associated endotheliopathy (SARS-CoV-2)	Pathogen-induced endotheliopathy (SARS-CoV-2)
		Vaccine-associated endotheliopathy (SARS-CoV-2 vaccines)
Activated hemostatic path	ULVWF path via damaged ECs	ULVWF path via damaged ECs
Involved vessels	Capillaries/arterioles	Veins/venules
Underlying thrombophlia	ADAMTS13 insufficiency	ADAMTS13 insufficiency(?)
Endothelial pathogenesis	Complement activation	Complement activation
**Phenotype and marker**		
Thrombosis character	Arterial microvascular microthrombi	Venous microthrombi
Clinical phenotype	TTP-like syndrome	ITP-like syndrome (“Silent” ITP)
	Consumptive thrombocytopenia	Consumptive thrombocytopenia
	MAHA	MOIS
	MODS/MOIS	
Endothelial markers	ULVWF ( ↑ VWF antigen ↑ FVIII activity)	ULVWF (↑ VWF antigen ↑ FVIII activity)
**Secondary pathogenesis**		
Cause of additional vascular injury	From arterial vascular access in hospital	From venous vascular access in hospital
	From arterial vascular injury outside hospital	From incidental head injury (?) after vaccine (e.g., CVST)
Activated hemostatic path	TF path via damaged SET	TF path via damaged SET
Affected vessels	Terminal arterial trees	Localized veins at injury site
Molecular phenotype	Microthrombi and fibrin meshes	Microthrombi and fibrin meshes
Pathologic phenotype	Numerous, minute macrothrombi shower composed of microthrombi and fibrin meshes in arterioles and capillaries in the digits, producing well-demarcated peripheral gangrene	Large, multiple, irregular, connected macrothrombi made of microthrombi and fibrin meshes in local or regional veins, producing DVT complex and venous stasis
**Clinical syndrome**	Arterial micro-macrothrombosis (Gangrene syndrome)	Venous micro-macrothrombosis (complex DVT syndrome)
Examples of syndromes	SPG, PF, ANF, diabetic gangrene, limb gangrene, Fournier’s disease, Burger’s disease, acrocyanosis)	VTE, CVST, IVCT, PTE, BCS, PVT, SVCT, SVT

Abbreviations: aEA-VMTD, arterial endotheliopathy-associated vascular microthrombotic disease; vEA-VMTD, venous-VMTD; ANF, acute necrotizing fasciitis; BCS, Budd-Chiari syndrome; CVST, cerebral venous sinus thrombosis; ECs, endothelial cells, DVT, deep venous thrombosis; IVCT, inferior vena cava thrombosis; PF, purpura fulminans; PTE, pulmonary thromboembolism; PVT, portal vein thrombosis; SET, subendothelial issue; SPG, symmetrical peripheral gangrene; SVCT, superior vena cava thrombosis; SVT, splanchnic vein thrombosis; TF, tissue factor; PTE, pulmonary thromboembolism; ULVWF, unusually large von Willebrand factor: VTE, venous thromboembolism.

**Table 4:** Diagnostic clinical and laboratory findings due to combined micro-macrothrombosis in aEA-VMTD and vEA-VMTD.

Combined Micro-Macrothrombosis due to aEA-VMTD (Gangrene Syndromes) (e.g., Sepsis)	Combined Micro-Macrothrombosis due to vEA-VMTD (Proximal/Central DVT) (e.g., Sepsis or after Vaccination)
**Clinical features**	**Clinical features**
Fever/fatigue/myalgia	Fever/fatigue/myalgia
TTP-like syndrome with thrombocytopenia and MAHA	ITP-like syndrome (“Silent” ITP)
MODS (e.g., ARDS; encephalopathy)	MOIS (e.g., myocarditis; pericarditis)
Gangrene syndromes (e.g., SPG; limb gangrene)	VTE (e.g., VTE; CVST; PTE; SVT)
“Gangrene”	“Venous congestion syndrome”
**Laboratory changes (demonstrated)**	**Laboratory changes (demonstrated or expected)**
Endothelial (ULVWF path) markers	Endothelial (ULVWF path) markers
Consumptive thrombocytopenia	Consumptive thrombocytopenia
Overexpressed ULVWF/VWF antigen	Overexpressed ULVWF/VWF antigen
Increased FVIII activity	Increased FVIII activity
Increased thrombomodulin	Increased thrombomodulin
Endothelial epiphenomenon	Endothelial epiphenomenon
Positive ANA	Positive ANA
Positive anti-dsDNA	Positive anti-dsDNA
Positive anti-PL antibodies	Positive anti-PL antibodies
Positive PF4 antibodies	Positive PF4 antibodies
Tissue factor path markers	Tissue factor path markers
Positive D-dimer	Positive D-dimer
TF-bearing microvesicles in circulation	TF-bearing microvesicles in circulation (expected)

Abbreviation: ANA, antinuclear antibodies; CVST, cerebral venous sinus thrombosis; aEA-VMTD, arterial endotheliopathy-associated vascular micro-thrombotic disease; vEA-VMTD, venous EA-VMTD; MODS, multiorgan dysfunction syndrome; MOIS, multi-organ inflammatory syndrome; ITP, immune thrombocytopenic purpura; PF4, platelet factor 4; PL, phospholipids; PTE, pulmonary thromboembolism; SPG, symmetrical peripheral gangrene; SVT, splanchnic vein thrombosis; TTP, thrombotic thrombocytopenic purpura; VTE, venous thromboembolism.

## Data Availability

Data sharing not applicable to this article as no datasets were generated or analyzed during the current study.

## Supplementary Materials

The following are available online at https://www.mdpi.com/article/10.3390/medicina57111163/s1, Table S1: Examples of organ inflammatory syndrome observed following vaccination (neither proven nor disproven in most of cases).
