# Supplementary material for: Vaccine-Associated Thrombocytopenia and Thrombosis: Venous Endotheliopathy Leading to Venous Combined Micro-Macrothrombosis
Source: Medicina (Kaunas). 2021 Oct 26;57(11):1163. doi: 10.3390/medicina57111163 (PMC8621006; doi:10.3390/medicina57111163)
Supplement: Supplementary file 1 [file medicina-57-01163-s001.zip › medicina-1416054-supplementary.pdf]

Supplementary 1

Table S1. Examples of organ inflammatory syndrome observed following vaccination (neither proven nor disproven in most of cases).

Organ Examples	Inflammatory Organ Syndromes	Cause-Effect Relationship Established	Sub-References	
			Non-COVID-19 Era	COVID-19 Era
CNS system	Encephalitis		[1,2]	[3,4]
Peripheral nerve	Guillain Barre syndrome	Yes	[5,6]	[7,8]
Thyroids	Subacute thyroiditis/Graves' disease		[9,10]	[11,12]
Lymph system	Lymphadenopathy/lymphadenitis		[13,14]	[15,16]
Pancreas	Acute pancreatitis		[17,18]	[19,20]
Liver	Hepatitis		[21,22]	[23,24]
Kidneys	Glomerulonephritis/IgA nephropathy		[25,26]	[27,28]
Adrenals	ASIA/adrenalitis		[29,30]	[31,32]
Muscles	Myositis		[33,34]	[35,36]
Heart	Myocarditis/pericarditis	Yes	[37,38]	[39,40]

Abbreviations: ASIA, autoimmune/inflammatory syndrome by adjuvants; CNS central nervous system. Sub-references from [1] to [40]: see Supplementary Materials.

Sub-references for Table S1

1 Doja A. Genetics and the myth of vaccine encephalopathy. *Paediatr Child Health*. 2008;13(7):597-599.

2 Mäkelä A, Nuorti JP, Peltola H. Neurologic disorders after measles-mumps-rubella vaccination. *Pediatrics*. 2002;110(5):957-963.

3 Torrealba-Acosta G, Martin JC, Huttenbach Y, et al. Acute encephalitis, myoclonus and Sweet syndrome after mRNA-1273 vaccine. *BMJ Case Rep*. 2021;14(7):e243173.

4 Vogrig A, Janes F, Gigli GL, et al. Acute disseminated encephalomyelitis after SARS-CoV-2 vaccination. *Clin Neurol Neurosurg*. 2021;208:106839.

5 Haber P, Sejvar J, Mikaeloff Y, DeStefano F. Vaccines and Guillain-Barré syndrome. *Drug Saf*. 2009;32(4):309-323.

6 Souayah N, Nasar A, Suri MF, Qureshi AI. Guillain-Barre syndrome after vaccination in United States a report from the CDC/FDA Vaccine Adverse Event Reporting System. *Vaccine*. 2007;25(29):5253-5255.

7 Maramattom BV, Krishnan P, Paul R, et al. Guillain-Barré Syndrome following ChAdOx1-S/nCoV-19 Vaccine. *Ann Neurol*. 2021;90(2):312-314.

8 Allen CM, Ramsamy S, Tarr AW, et al. Guillain-Barré Syndrome Variant Occurring after SARS-CoV-2 Vaccination. *Ann Neurol*. 2021;90(2):315-318.

9 Watad A, David P, Brown S, Shoenfeld Y. Autoimmune/Inflammatory Syndrome Induced by Adjuvants and Thyroid Autoimmunity. *Front Endocrinol (Lausanne)*. 2017;7:150.

10 Girgis CM, Russo RR, Benson K. Subacute thyroiditis following the H1N1 vaccine. *J Endocrinol Invest*. 2010;33(7):506.
11 İremli BG, Şendur SN, Ünlütürk U. Three Cases of Subacute Thyroiditis Following SARS-CoV-2 Vaccine: Post-vaccination ASIA Syndrome [published online ahead of print, 2021 May 27]. *J Clin Endocrinol Metab*. 2021;dgab373.
12 Domiguez MP, Medina G, Sánchez Valadez TI, Jara LJ. Two Cases of Graves' Disease Following SARS-CoV-2 Vaccination: An Autoimmune/Inflammatory Syndrome Induced by Adjuvants. *Thyroid*. 2021;10.1089/thy.2021.0142.
13 Hartsock RJ, Bellanti JA. Postvaccinial lymphadenitis. *GP*. 1969;39(1):99-105.
14 Aelami MH, Alborzi A, Pouladfar G, Geramizadeh B, Pourabbas B, Mardaneh J. Post-Vaccination Disseminated Bacillus Calmette Guerin Infection Among Children in Southern Iran. *Jundishapur J Microbiol*. 2015;8(11):e25663
15 Keshavarz P, Yazdanpanah F, Rafiee F, Mizandari M. Lymphadenopathy Following COVID-19 Vaccination: Imaging Findings Review. *Acad Radiol*. 2021;28(8):1058-1071.
16 Hiller N, Goldberg SN, Cohen-Cymberknoh M, Vainstein V, Simanovsky N. Lymphadenopathy Associated With the COVID-19 Vaccine. *Cureus*. 2021;13(2):e13524.
17 Adler JB, Mazzotta SA, Barkin JS. Pancreatitis caused by measles, mumps, and rubella vaccine. *Pancreas*. 1991;6(4):489-490.
18 Bizjak M, Bruck O, Praprotnik S, Dahan S, Shoenfeld Y. Pancreatitis after human papillomavirus vaccination: a matter of molecular mimicry. *Immunol Res*. 2017;65(1):164-167.
19 Parkash O, Sharko A, Farooqi A, Ying GW, Sura P. Acute Pancreatitis: A Possible Side Effect of COVID-19 Vaccine. *Cureus*. 2021;13(4):e14741.
20 Cieślewicz A, Dudek M, Krela-Kaźmierczak I, Jabłecka A, Lesiak M, Korzeniowska K. Pancreatic Injury after COVID-19 Vaccine-A Case Report. *Vaccines (Basel)*. 2021;9(6):576.
21 van Gemeren MA, van Wijngaarden P, Doukas M, de Man RA. Vaccine-related autoimmune hepatitis: the same disease as idiopathic autoimmune hepatitis? Two clinical reports and review. *Scand J Gastroenterol*. 2017;52(1):18-22.
22 Sasaki T, Suzuki Y, Ishida K, et al. Autoimmune hepatitis following influenza virus vaccination: Two case reports. *Medicine (Baltimore)*. 2018;97(30):e11621.
23 Rela M, Jothimani D, Vij M, Rajakumar A, Rammohan A. Auto-immune hepatitis following COVID vaccination. *J Autoimmun*. 2021;123:102688.
24 Mann R, Sekhon S, Sekhon S. Drug-Induced Liver Injury After COVID-19 Vaccine. *Cureus*. 2021;13(7):e16491.
25 Tan SY, Cumming AD. Vaccine related glomerulonephritis. *BMJ*. 1993;306(6872):248.
26 Patel C, Shah HH. Vaccine-associated kidney diseases: A narrative review of the literature. *Saudi J Kidney Dis Transpl*. 2019;30(5):1002-1009.
27 Abramson M, Mon-Wei Yu S, Campbell KN, Chung M, Salem F. IgA Nephropathy After SARS-CoV-2 Vaccination. *Kidney Med*. 2021;10.1016/j.xkme.2021.05.002.
28 Hanna C, Herrera Hernandez LP, Bu L, et al. IgA nephropathy presenting as macroscopic hematuria in 2 pediatric patients after receiving the Pfizer COVID-19 vaccine. *Kidney Int*. 2021;100(3):705-706.
29 Bragazzi NL, Hejly A, Watad A, Adawi M, Amital H, Shoenfeld Y. ASIA syndrome and endocrine autoimmune disorders. *Best Pract Res Clin Endocrinol Metab*. 2020;34(1):101412.
30 Kamath S, Khabra JK, Desai P, Frunzi J. Adrenal Crisis Secondary to Influenza and Tetanus Vaccination in an Adult Without Known Adrenal Insufficiency: A Case of Autoimmune Adrenalitis. *Cureus*. 2021;13(7):e16312.

31 Varona JF, García-Isidro M, Moeinvaziri M, Ramos-López M, Fernández-Domínguez M. Primary adrenal insufficiency associated with Oxford-AstraZeneca ChAdOx1 nCoV-19 vaccine-induced immune thrombotic thrombocytopenia (VITT). *Eur J Intern Med*. 2021;91:90-92.
32 Freire Santana M, Borba MGS, Baía-da-Silva DC, et al. Case Report: Adrenal Pathology Findings in Severe COVID-19: An Autopsy Study. *Am J Trop Med Hyg*. 2020;103(4):1604-1607.
33 Basilious A, Jivraj I, DeAngelis D. Acute Unilateral Ptosis and Myositis Following the H1N1 Influenza Vaccine. *Ophthalmic Plast Reconstr Surg*. 2020;36(1):e16-e17.
34 Gherardi RK, Crépeaux G, Authier FJ. Myalgia and chronic fatigue syndrome following immunization: macrophagic myofasciitis and animal studies support linkage to aluminum adjuvant persistency and diffusion in the immune system. *Autoimmun Rev*. 2019;18(7):691-705.
35 Theodorou DJ, Theodorou SJ, Axiotis A, Gianniki M, Tsifetaki N. COVID-19 vaccine-related myositis [published online ahead of print, 2021 Feb 27]. *QJM*. 2021;hcab043.
36 Godoy IRB, Rodrigues TC, Skaf A. Myositis ossificans following COVID-19 vaccination [published online ahead of print, 2021 Jun 9]. *QJM*. 2021;hcab161.
37 Dalgaard JB. Fatal myocarditis following smallpox vaccination. *Am Heart J*. 1957;54(1):156-157.
38 Mei R, Raschi E, Forcesi E, Diemberger I, De Ponti F, Poluzzi E. Myocarditis and pericarditis after immunization: Gaining insights through the Vaccine Adverse Event Reporting System. *Int J Cardiol*. 2018;273:183-186.
39 Bozkurt B, Kamat I, Hotez PJ. Myocarditis With COVID-19 mRNA Vaccines. *Circulation*. 2021;144(6):471-484.
40 Laganà N, Cei M, Evangelista I, et al. Suspected myocarditis in patients with COVID-19: A multicenter case series. *Medicine (Baltimore)*. 2021;100(8):e24552.
